# Cardiac evaluation in amiodarone-induced thyroid dysfunction with suspected cardiac ischemia?: a case report and review of the literature

**DOI:** 10.1186/s13256-024-04552-w

**Published:** 2024-05-02

**Authors:** Yoann Aubry, Michel Dosch, Marc Y. Donath

**Affiliations:** 1 Clinic of Endocrinology, Diabetes and Metabolism, Hospital Delémont, Hôpital du Jura, Faubourg Des Capucins 30, 2800 Delémont, Switzerland; 2 Clinic of Endocrinology, Diabetes and Metabolism, University Hospital Basel, Basel, Switzerland; 3 The Division of Digestive Surgery, Surgery Department, University Hospitals of Geneva, Geneva, Switzerland

**Keywords:** Hyperthyroidism, Amiodarone, Cardiac ischemia, Thyroidectomy, Case report

## Abstract

**Background:**

Amiodarone-induced thyroid dysfunction (AIT) is a side-effect associated with the use of Amiodarone for the treatment of refractory arrythmias. Resulting hyperthyroidism can precipitate cardiac complications, including cardiac ischemia and myocardial infarction, although this has only been described in a few case reports.

**Case presentation:**

We present here a clinical scenario involving a 66-year-old male Caucasian patient under Amiodarone for atrial fibrillation, who developed AIT. In the presence of dyspnea, multiple cardiovascular risk factors and ECG abnormalities, a transthoracic echocardiogram was performed, showing inferobasal hypokinesia. This led to further investigations through a cardiac PET-CT, where cardiac ischemia was suspected. Ultimately, the coronary angiography revealed no abnormalities. Nonetheless, these extensive cardiologic investigations led to a delay in initiating an emergency endovascular revascularization for acute-on-chronic left limb ischemia. Although initial treatment using Carbimazole was not successful after three weeks, the patient reached euthyroidism after completion of the treatment with Prednisone so that eventually thyroidectomy was not performed. Endovascular revascularization was finally performed after more than one month.

**Conclusions:**

We discuss here cardiac abnormalities in patients with AIT, which may be due to relative ischemia secondary to increased metabolic demand during hyperthyroidism. Improvement of cardiac complications is expected through an optimal AIT therapy including medical therapy as the primary approach and, when necessary, thyroidectomy. Cardiac investigations in the context of AIT should be carefully considered and may not justify delaying other crucial interventions. If considered mandatory, diagnostic procedures such as coronary angiography should be preferred to functional testing.

## Introduction

Amiodarone is a class III anti-arrhythmic drug widely used in clinical practice as second-line therapy for refractory ventricular and supra-ventricular arrythmias [1]. Thyroid dysfunction under amiodarone affects up to 20% of treated patients, either hyperthyroidism/thyrotoxicosis or hypothyroidism [2, 3]. Two types of Amiodarone-Induced Thyroid Dysfunction (AIT) are distinguished: type 1 AIT is a form of iodine-induced hyperthyroidism typically arising in a gland with underlying functional autonomy, while type 2 AIT is a destructive thyroiditis developing in a normal gland. The treatment of choice usually consists of a combination of thionamides and glucocorticoids, as distinguishing between the two types of AIT can be challenging [2, 4, 5]. However, resolution of hyperthyroidism takes usually several weeks to months.

Amiodarone-induced hyperthyroidism may be associated to serious cardiac complications. In particular, hyperthyroidism can lead to cardiac ischemia and even to a secondary myocardial infarction [6–12]. In addition, hyperthyroidism is also associated with a pro-coagulant state and can aggravate cardiac and limb ischemia [13, 14]. Both conditions might benefit from an early thyroidectomy.

Therefore, one challenge resides in the indication and the optimal timing for the thyroidectomy. Indeed, anaesthetizing a patient with a decreased hemodynamic status or suspicion for cardiac ischemia can be challenging and the need for further investigations with the aim to mitigate potential intra- or peri-operative complications should be balanced with the delay of the thyroidectomy.

We describe here a clinical scenario with AIT, suspected cardiac ischemia and acute-on-chronic limb ischemia. Extensive cardiac investigations were carried out, which revealed false-positive functional imaging, probably due to increased cardiac metabolism, resulting in a delay in revascularization of limb ischemia. Our aim is to discuss the rationale of such investigations and the benefits of an early thyroidectomy.

## Materials and methods

In accordance with ethical guidelines, written informed consent was obtained from the patient involved for the publication of this case report and any accompanying images. A copy of the written consent is available for review by the Editor-in-Chief of this journal. The patient's identity has been rigorously protected, and any potentially identifying information has been appropriately anonymized to ensure confidentiality and privacy.

In order to discuss the association between hyperthyroidism and cardiac ischemia, and the indication for cardiac investigations in AIT, we conducted a literature research on PubMed and MEDLINE using the following keywords: “Amiodarone-Induced Thyroid Dysfunction”, “hyperthyroidism”, “cardiac ischemia”, “thyroidectomy”. Only articles written in English were considered for this case report.

## Case presentation

Our patient is a 66-years old male Caucasian patient known for active smoking (~ 60 pack years), hypertension and dyslipidemia, who presented with severe hyperthyroidism induced by amiodarone (given for the treatment of atrial fibrillation). At the time of diagnosis, he presented with tachycardia but no hemodynamic instability, NYHA stage 2 dyspnea and unexplained weight loss. Initial laboratory work-up showed suppressed TSH < 0.05 mUI/l, free T3 13.9 pmol/l and free T4 72 pmol/l. Anti-TSH antibodies were negative. Thyroid ultrasound showed a hypovascular thyroid and the absence of thyroid nodules. Thyreostatic treatment with Carbimazole tablets 60 mg/day was initiated. After 3 weeks, the patient was still hyperthyroid, so prednisone tablets 40 mg/day were added.

Meanwhile, the patient displayed obliterative arteriopathy of the lower limbs, stage IIA Fontaine in the left lower limb, with distal embolization of a tight stenosis of the homolateral common iliac artery, stage I Fontaine in the right lower limb. Although left limb ischemia was not critical, it required a rapid endovascular revascularization. Thyroid normalization is essential prior to vascular intervention, due to the procoagulant state associated with hyperthyroidism.

**Fig. 1:**
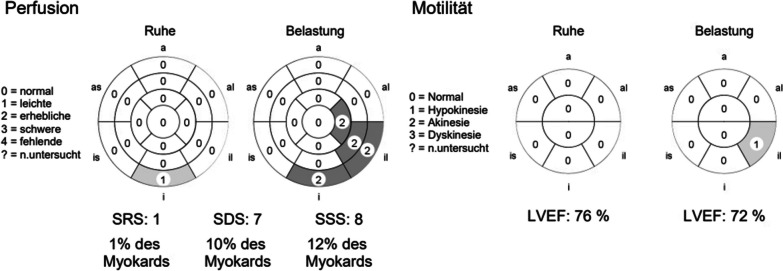
Cardiac PET scan findings of our patient. No scar but evidence of infero-lateral to infero-apical ischemia (Perfusion). Localized decreased flow reserve in ischemia area. Preserved left ventricular pump function (Motilität) with normal left ventricular ejection fraction (LVEF). *SRS* summed rest score, *SDS* summed difference score, *SSS* summed stress score, *a* apical, *as* apico-septal, *al* apico-lateral, *is* infero-septal, *il* infero-lateral, *i* inferior

In the absence of response to drug treatment after three weeks, thyroidectomy was discussed. In this context, and due to a high cardiovascular risk associated with dyspnea, a transthoracic echocardiogram showed inferobasal hypokinesia and a cardiac PET-CT was performed, showing suspected inferobasal and inferoapical ischemia (Fig. 1).

After multidisciplinary discussion involving cardiology, anesthesiology, angiology and endocrinology, it was decided to perform coronary angiography as soon as possible to rule out cardiac ischemia. Coronary angiography showed no abnormalities requiring revascularization.

Following the various cardiac investigations and the related delay, the evolution of his thyroid function was resolved after more than a month (TSH 0.91mUI/l, free T4 14.2pmol/l, free T3 3.2pmol/l) without surgery, and the patient underwent successful endovascular revascularisation for his peripheral arterial disease.

## Discussion

The clinical scenario presented here emphasizes a patient exhibiting amiodarone-induced hyperthyroidism, requiring prompt intervention to mitigate potential complications, including acute limb ischemia. However, concerns regarding cardiac ischemia introduced a slowdown in the treatment process. Ultimately, thyroidectomy was not performed and vascular interventions were postponed until euthyroid status was achieved. In this situation, where the treatment of hyperthyroidism and peripheral arterial disease are decisive, it is of interest to discuss the indication for additional cardiologic investigations, including cardiac PET scan. Especially considering the impact of hyperthyroidism on cardiac muscle [15, 16], and the recent literature suggesting that patients with hyperthyroidism-related cardiac complications might benefit from an early thyroidectomy [17–20]. Indeed, PET scan in patients with hyperthyroidism may display false positive results for coronary artery disease due to hypermetabolism with consecutive relative ischemia.

AIT can be associated with cardiovascular complications, including rhythm disorders such as tachycardia and atrial fibrillation, along with high-output congestive heart failure, among others [15, 16]. In more uncommon instances, it may also result in cardiac ischemia and acute myocardial infarction [6–12].

Thyroid hormones induce cell proliferation in the vascular wall typically leading to cardiac hypertrophy with progressively worsening high-output congestive heart failure, as evidenced by a decrease in echocardiography-measured left ventricular ejection fraction (LVEF) [21].

Cardiac Ischemia related to hyperthyroidism is rarely described in the literature and is the topic of older publications and a limited number of case reports [6–12]. It has been particularly described in elderly patients with coronary artery disease [22]. The mechanisms explaining cardiac ischemia in patients with hyperthyroidism remain unclear, but they might involve an increased myocardial oxygen demand related to cardiac hypertrophy, temporary coronary artery occlusion in a procoagulant state and vasoconstriction / vasospasm. Although the molecular mechanism remains unclear, there is some similarity with the clinical impact of excess catecholamines on the suffering of cardiac muscle. This may be due to thyroid hormones, which enhance the sympathetic nervous system by increasing the sensitivity of beta receptors [23]*.*

Indeed, hyperthyroidism is associated with a procoagulant state disrupting both primary and secondary hemostasis. Among other factors, elevation of von Willebrand factor levels with enhanced platelets function and increased factor X activity in the coagulation cascade have been described, all contributing to an elevated risk of coronary artery disease [13, 14]. In addition, the increase of thyroid hormones seem to be associated to coronary vascular degeneration and plaque instability [24]. In our case, this also justify to treat the limb ischemia in euthyroidism.

Thyroid hormones are also known to sensitize adrenergic receptors what enhances the vasoconstrictor effect of catecholamines on coronary arteries and hyperkinetic circulation, another possible cause of cardiac ischemia [16].

The clinical presentation and concomitant medications suggest AIT. However, the exact etiology of hyperthyroidism remains unclear and amiodarone-induced destructive thyroiditis is conceivable.

Given cardio-vascular complications related to hyperthyroidism, patients will primarily benefit from prompt treatment and restoration of euthyroidism [21]. Antithyroid drugs are the first-line treatment in this case, usually a combination of thionamides and glucocorticoids [4, 5]. Discontinuing amiodarone, which has a half-life of 100 days and acts as a T4 to T3 conversion inhibitor, is not recommended [2, 4, 5]. It will likely be ineffective and, even worse, might exacerbate the thyrotoxic state, potentially resulting in dangerous arrhythmias. Since medical treatment is expected to improve cardiac symptoms [12], additional cardiac investigations, such as a cardiac PET scan or a coronary angiography, should always be critically evaluated as they could potentially delay other critical interventions. A cardiac PET scan can suspect cardiac ischemia related for example to vasospasm in the coronary angiography, which can be resolved with appropriately treated hyperthyroidism [6–8, 11, 25]. However, a coronary angiography remains clearly indicated in case an acute myocardial infarction is suspected.

In line with the guidelines of the European Thyroid Association (ETA), a total or near-total thyroidectomy should then be considered for patients who do not respond to medical treatment for more than 2 weeks, exhibit a worsening cardiac status, or experience severe thyrotoxicosis requiring prompt resolution [20, 26]. In our case, rapid resolution of hyperthyroidism was necessary to perform endovascular limb revascularization.

Although anaesthetizing a patient with hyperthyroidism or AIT can be challenging, it doesn’t appear to be associated with increased intra- or peri-operative complications [17, 27–29]. However, one study described slightly elevated peri-operative morbidity and mortality [20]. Although patients are usually operated in euthyroid state, it has been shown in recent publications that patients with a mild to severe heart insufficiency for example will benefit from early thyroidectomy, in thyrotoxicosis when necessary [17–20]. This approach also appears reasonable, particularly when other critical operations are necessary, as in this clinical scenario a rapid endovascular revascularization. In our case, adopting a watchful-waiting strategy regarding the operation could have been associated to increased risk for complications, including exacerbation of limb ischemia.

## Conclusions

Cardiac investigations with metabolic imaging (e.g., PET scan) in the context of AIT should not delay thyroidectomy especially when other crucial interventions are planned. A coronary angiography remains indicated in case of high suspicion of coronary stenosis or myocardial infarction. Thyroid surgery should be considered early in the treatment plan and patients might benefit from a simplified procedure.

## Data Availability

Data supporting our findings were taken from the patient’s folder.
